# Physicochemical characteristics of chitosan from swimming crab (*Portunus trituberculatus*) shells prepared by subcritical water pretreatment

**DOI:** 10.1038/s41598-021-81318-0

**Published:** 2021-01-18

**Authors:** Gengxin Hao, Yanyu Hu, Linfan Shi, Jun Chen, Aixiu Cui, Wuyin Weng, Kazufumi Osako

**Affiliations:** 1 College of Food and Biological Engineering, Jimei University, Xiamen, 361021 China; 2 Engineering Research Center of the Modern Technology for Eel Industry, Ministry of Education, Xiamen, 361021 China; 3 Department of Food Science and Technology, Tokyo University of Marine Science and Technology, Tokyo, 108-8477 Japan

**Keywords:** Biological techniques, Ocean sciences

## Abstract

The physicochemical properties of chitosan obtained from the shells of swimming crab (*Portunus trituberculatus*) and prepared via subcritical water pretreatment were examined. At the deacetylation temperature of 90 °C, the yield, ash content, and molecular weight of chitosan in the shells prepared via subcritical water pretreatment were 12.2%, 0.6%, and 1187.2 kDa, respectively. These values were lower than those of shells prepared via sodium hydroxide pretreatment. At the deacetylation temperature of 120 °C, a similar trend was observed in chitosan molecular weight, but differences in chitosan yield and ash content were not remarkable. At the same deacetylation temperature, the structures of chitosan prepared via sodium hydroxide and subcritical water pretreatments were not substantially different. However, the compactness and thermal stability of chitosan prepared via sodium hydroxide pretreatment was lower than those of chitosan prepared via subcritical water pretreatment. Compared with the chitosan prepared by sodium hydroxide pretreatment, the chitosan prepared by subcritical water pretreatment was easier to use in preparing oligosaccharides, including (GlcN)_2_, via enzymatic hydrolysis with chitosanase. Results suggested that subcritical water pretreatment can be potentially used for the pretreatment of crustacean shells. The residues obtained via this method can be utilized to prepare chitosan.

Swimming crab (*Portunus trituberculatus*) is an economically important aquatic species widely distributed in the coastal waters of China, Japan, and Korea^1^. The annual catch of swimming crab in China was approximately 458 million tons in 2019^2^. Swimming crab is mainly used to produce crab meat can products. During crab meat can processing, the body and claw meat are manually removed from cooked crabs, resulting in huge amounts of crab shell waste containing meat protein. Needless to say, the meat protein should be recovered and chitin/chitosan should be extracted from crab by-products. In a previous study, the effects of subcritical water temperature (140–230 °C) on the properties of swimming crab shell extracts containing meat protein were investigated. Proteins from crab shell could be extracted at subcritical water temperature at 170 °C, and the amino acids in the extracts were not destroyed^3^.

Subcritical water (with high H_3_O^+^ and OH^−^ concentrations) was pressurized at temperatures from 100 to 374 °C; thus, it can serve as an extraordinary solvent for various cellulosic and proteinaceous biomass^4–7^. Subcritical water can promote α-chitin decomposition at 260–320 °C, and (GlcNAc)_2_ can be produced by crab shell treatment at 350 °C for 7 min followed by enzymatic degradation; for example, this treatment weakens hydrogen bonds^8^. Under high subcritical water temperatures, chitin is hydrolyzed and oligosaccharides, including (GlcNAc)_2_ and GlcNAc, are decomposed^9,10^. Acidified subcritical water can extract quickly mineral elements including calcium, and citric acid is widely used to product commercial food as food-grade organic acid^11,12^. However, the physicochemical properties of chitin/chitosan from crab shells prepared by subcritical water pretreatment at a relatively low temperature (170 °C) have not been investigated thus far.

Traditional chitin prepared from crustacean shells involves the use of strong acids and bases for demineralization and deproteinization^13^. The proteins removed by strong bases cannot be further applied, and disposal of deproteinized sewage results in serious environmental pollution. Moreover, the traditional deacetylation for preparing chitosan from chitin also requires a solution with high alkali content (40–60%), leading to severe environmental pollution^14^. Owing to chitin shortage associated with the traditional preparation of crab shells, this study aimed to prepare chitosan from swimming crab shells via subcritical water pretreatment instead of alkali treatment. Another strategy applied herein was increasing the deacetylation temperature to decrease the alkali concentration for deacetylation. The ash content, deacetylation degree (DD), molecular weight, and UV absorption of the extracted chitosan were determined via Fourier transform infrared (FTIR) spectroscopy, X-ray diffraction (XRD) analysis, and microstructural and thermal stability tests. The results of the present study may aid in the development of new approaches for extracting chitosan/oligosaccharides from crustacean shells.

## Results and discussions

### Physiochemical parameters

Chitosan was prepared from swimming crab shells by applying different deproteinization and deacetylation methods. Various chitosan parameters, including yield, ash, DD, and molecular weight are shown in Table 1. At the deacetylation temperature of 90 °C, chitosan yield ranged from 12.0 to 13.2%, which was lower than that of chitosan from shrimp shells^15^. The yield of chitosan prepared using sodium hydroxide (chitosan A-90) was slightly higher than that of chitosan prepared using subcritical water (chitosan B-90 and chitosan C-90). A similar trend was observed in terms of ash content. When the deacetylation temperature was increased to 120 °C, chitosan yields and ash contents decreased. However, differences in the yields and ash contents of chitosans deacetylated at 120 °C were not significant. The most common elements in crustacean shells are Ca, Mg, Na, K, and Fe^16^. Pressure and temperature can break the element–matrix bonds, which might be promoted by the acidified subcritical water^11,17^. Therefore, subcritical water pretreatment destroyed the element-chelating ability of chitin, causing it to be easily demineralized in this study (Table 1).

**Table 1:** Physiochemical parameters of chitosan extracted from swimming crab shells.

	Yield (%)	Ash (%)	Deacetylation degree (%)	Molecular weight (kDa)
Chitosan A-90	13.2 ± 0.2^a^	0.8 ± 0.1^a^	62.2 ± 1.5^e^	1816.3 ± 51.7^a^
Chitosan B-90	12.2 ± 0.4^b^	0.6 ± 0.1^b^	70.8 ± 1.5^d^	1187.2 ± 42.1^b^
Chitosan C-90	12.0 ± 0.2^b^	0.6 ± 0.1^b^	73.2 ± 1.5^d^	953.3 ± 59.5^c^
Chitosan A-120	11.7 ± 0.3^c^	0.4 ± 0.1^c^	80.5 ± 1.3^c^	598.7 ± 76.1^d^
Chitosan B-120	11.3 ± 0.2^c^	0.3 ± 0.1^c^	84.1 ± 1.2^b^	445.2 ± 35.5^e^
Chitosan C-120	11.2 ± 0.3^c^	0.3 ± 0.1^c^	88.5 ± 1.5^a^	306.5 ± 32.3^f^

Values with different letters in the same column are significantly different at *p* < 0.05.

Data are expressed as mean ± standard deviation, n = 3.

The DD of chitosan prepared using NaOH at 90 °C and 120 °C were 62.2% and 80.5%, respectively, which increased after subcritical water pretreatment without or with citric acid. The opposite trend was observed in terms of the molecular weight of chitosan subjected to different pretreatments. DD and molecular weight affect the physicochemical and functional properties of chitosan^18,19^. Chitosan possesses various excellent functional properties, but its high molecular weight is a structural parameter that influences its solubility and limits its potential applications^20^. The molecular weight of chitosan was decreased by changing the conditions of deacetylation reaction. Subcritical water pretreatment increased the DD, but the effect was weaker than that of deacetylation temperature (Table 1).

### UV absorption

**Figure 1:**
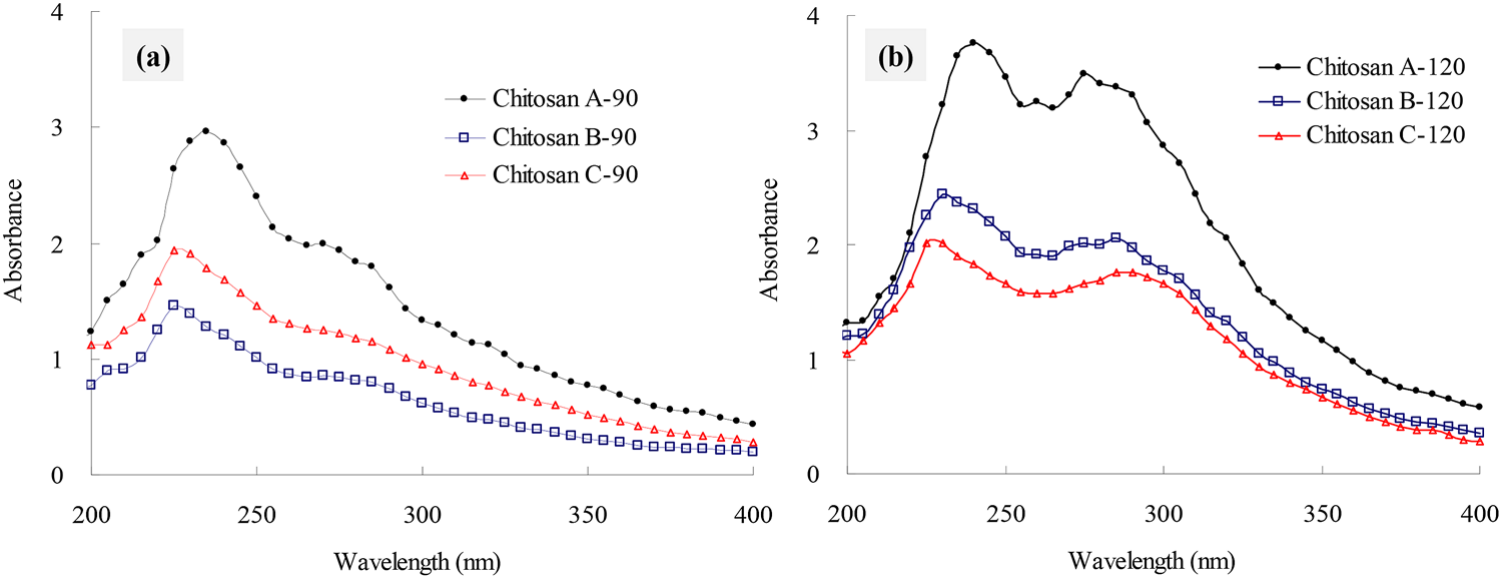
UV absorption spectra of crab shell chitosan by deacetylation at 90 °C (**a**) and 120 °C (**b**).

A characteristic peak at 235 nm could be due to n–π* transition; the absorption peak near 275 nm could be due to n → σ* transition for the amino groups; the spectral absorption at 300 nm was assigned to n → π* transition for the carbonyl or carboxyl groups^21,22^. When the swimming crab shells were deacetylated by sodium hydroxide at 90 °C, the chitosan A-90 obtained showed a sharp peak at approximately 235 nm with a shoulder peak near 275 nm (Fig. 1a). Compared with that of chitosan A-90, the sharp peak of chitosan B-90 blue-shifted to 225 nm, and the intensity of the shoulder peak near 275 nm decreased. This result suggested that the *N*-acetyl groups that stabilize the chitin structure in the crab shells was destroyed by subcritical water pretreatment at 170 °C. However, the changes between chitosan B-90 and chitosan C-90 pretreated using subcritical water with or without citric acid were negligible. When the deacetylation temperature increased to 120 °C, the UV absorption spectra of chitosan red-shifted by approximately 5 nm regardless of the deproteinization method. The intensities of the absorption peaks at 275 and 300 nm significantly increased (Fig. 1b) probably because the DD in chitosan was higher at 120 °C than at 90 °C.

### FTIR analysis

FTIR spectroscopy is an important tool for investigating the structure of chitin and chitosan^23^. The FTIR spectral bands at 3443–3416 cm^−1^ corresponded to OH– stretching and were assigned to the intramolecular hydrogen bonds –OH···OC–. The band at 3261 cm^−1^ characteristic to α-chitin corresponded to NH– stretching, which is involved in intermolecular and intramolecular hydrogen bonds^24^. Furthermore, the amide-I band in the α-chitin spectrum split at 1660 and 1625 cm^−1^, which were attributed to the intermolecular hydrogen bonds –CO···HO– and –CO···HN–, respectively^25^. The absorption bands at approximately 1560 and 1315 cm^−1^ were attributed to the chitosan characteristic peaks for amide II and amide III, respectively^26,27^.

**Figure 2:**
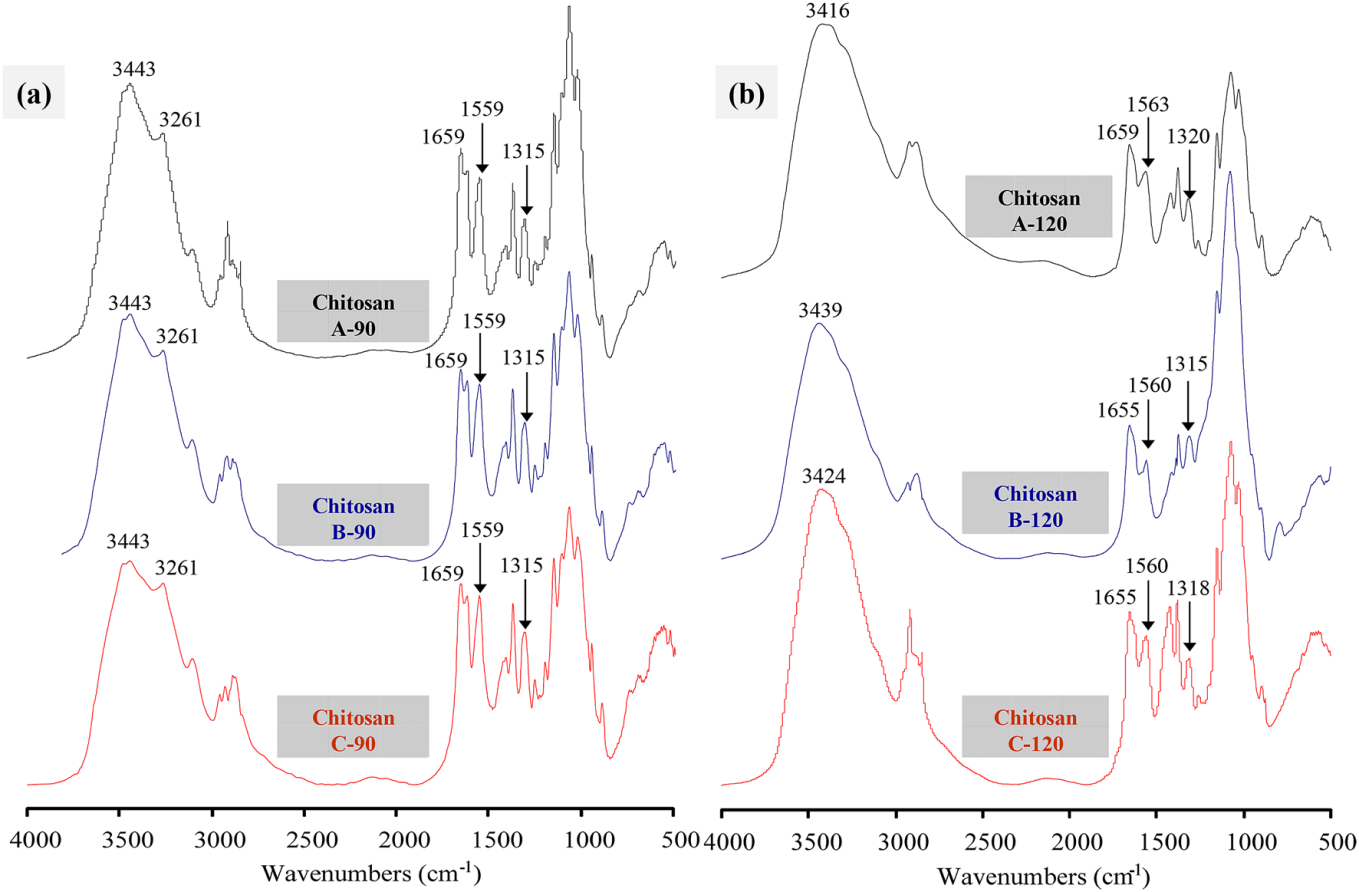
FTIR spectra of crab shell chitosan by deacetylation at 90 °C (**a**) and 120 °C (**b**).

The FTIR spectra of chitosan were determined to investigate the molecular structure of chitosan from swimming crab shells prepared via different methods (Fig. 2). According to the spectral bands at 3261 and 1315 cm^−1^, the chitosans prepared at the deacetylation temperature of 90 °C retained the characteristics of chitin, and these chitosans did not exhibit obvious differences. When the deacetylation temperature was increased to 120 °C, the characteristic peaks of chitin disappeared, and obvious changes were detected among the prepared chitosans in the spectral bands between 1400 and 1300 cm^−1^, which corresponded to OH– bending vibrations^28^. These results suggested that the structure of chitosan deacetylated at 120 °C was affected by the subcritical water pretreatment.

### XRD analysis

**Figure 3:**
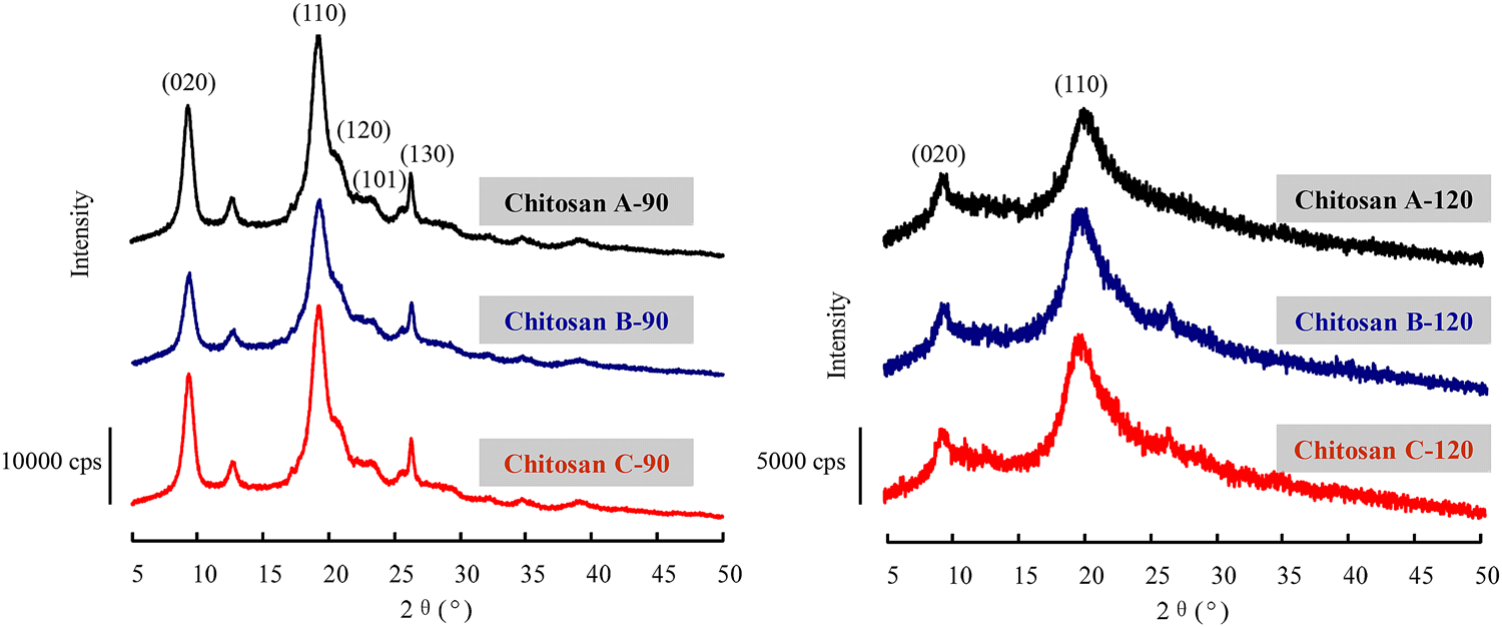
XRD patterns of crab shell chitosan by deacetylation at 90 °C and 120 °C.

The effects of deproteinization and deacetylation on the crystalline structures of chitosan were investigated via XRD within the 2θ range of 5°–50° (Fig. 3). When the deacetylation temperature was 90 °C, the three chitosans obtained had five crystalline reflections of 020, 110, 120, 101, and 130, corresponding to 2θ of 9.3°, 19.2°, 20.0°, 23.2°, and 26.3°, respectively. This result was consistent with the α-crystalline structures of chitins prepared from rice-field crab shells and cicada sloughs^29^. The peak intensity at 020 and 110 reflection reportedly decreases as DD increases^30^. A similar phenomenon was observed in the chitosan obtained from swimming crab shell and prepared at the deacetylation temperature of 90 °C. However, no obvious changes were observed in the reflection angle position of chitosan prepared via different deproteinization methods. By contrast, only the crystalline reflections of 020 and 110 were observed in the chitosans from crab shell prepared at the deacetylation temperature of 120 °C. Moreover, the two characteristic peaks were broader and had a lower intensity than those of chitosans prepared at the deacetylation temperature of 90 °C regardless of the deproteinization method. This result suggested that high deacetylation temperatures interrupted the hydrogen bonds within the structure of chitin, leading to lower crystallinity^31^. In general, the antiparallel chains in the chitin’s molecular structure favor the formation of strong intramolecular and intermolecular hydrogen bonds^20^. These hydrogen bonds hinder the rotation of neighboring sugar residues along glycoside bonds, thereby increasing their steric resistance and decreasing their rotation degree of freedom, ultimately enhancing crystallinity. As degreed increases, hydrogen bonds gradually weaken, and the chitosan molecules are less homogenous than the chitin molecules, a condition that is not conducive to the formation of a crystalline structure.

### Morphology

**Figure 4:**
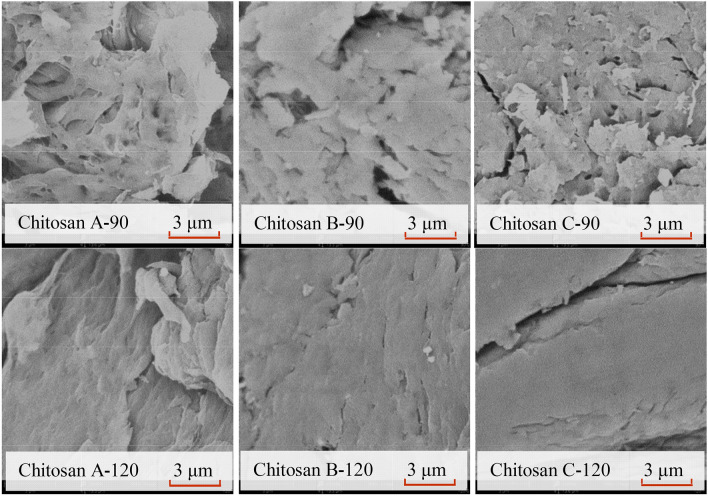
SEM images of crab shell chitosan by deacetylation at 90 °C and 120 °C.

The morphology of chitosan obtained from swimming crab shells was analyzed via SEM (Fig. 4). At the deacetylation temperature of 90 °C, loose and cotton-like fibers were observed on the surface of chitosan A-90. The cotton-like surface structure was more compact in chitosan B-90 than in chitosan A-90, whereas the structure was smaller in chitosan C-90. When the deacetylation temperature increased from 90 to 120 °C, the fiber structure on the chitosan surface became smoother and denser than that of chitosan B-90 regardless of deproteinization method. The tightness and uniformity of chitosan surface were enhanced for chitosan A-120, B-120, and C-120 (Fig. 4), similar to the reported surface morphology of chitosan with DD of 85% and 88%^32^. A similar phenomenon was observed in the micrographs of α-chitin as DD increased^33^. According to the results shown in Figs. 3 and 4, the surface morphology of chitosan is related to its crystal structure. At the same deacetylation temperature, differences in the surface morphology and crystal structure of chitosan molecules were not significant. The crystallinity between chitosan molecules sharply declined when the deacetylation temperature increased from 90 to 120 °C, resulting in the transformation of the flocculent aggregates into uniformly distributed blocks.

### DSC analysis

**Figure 5:**
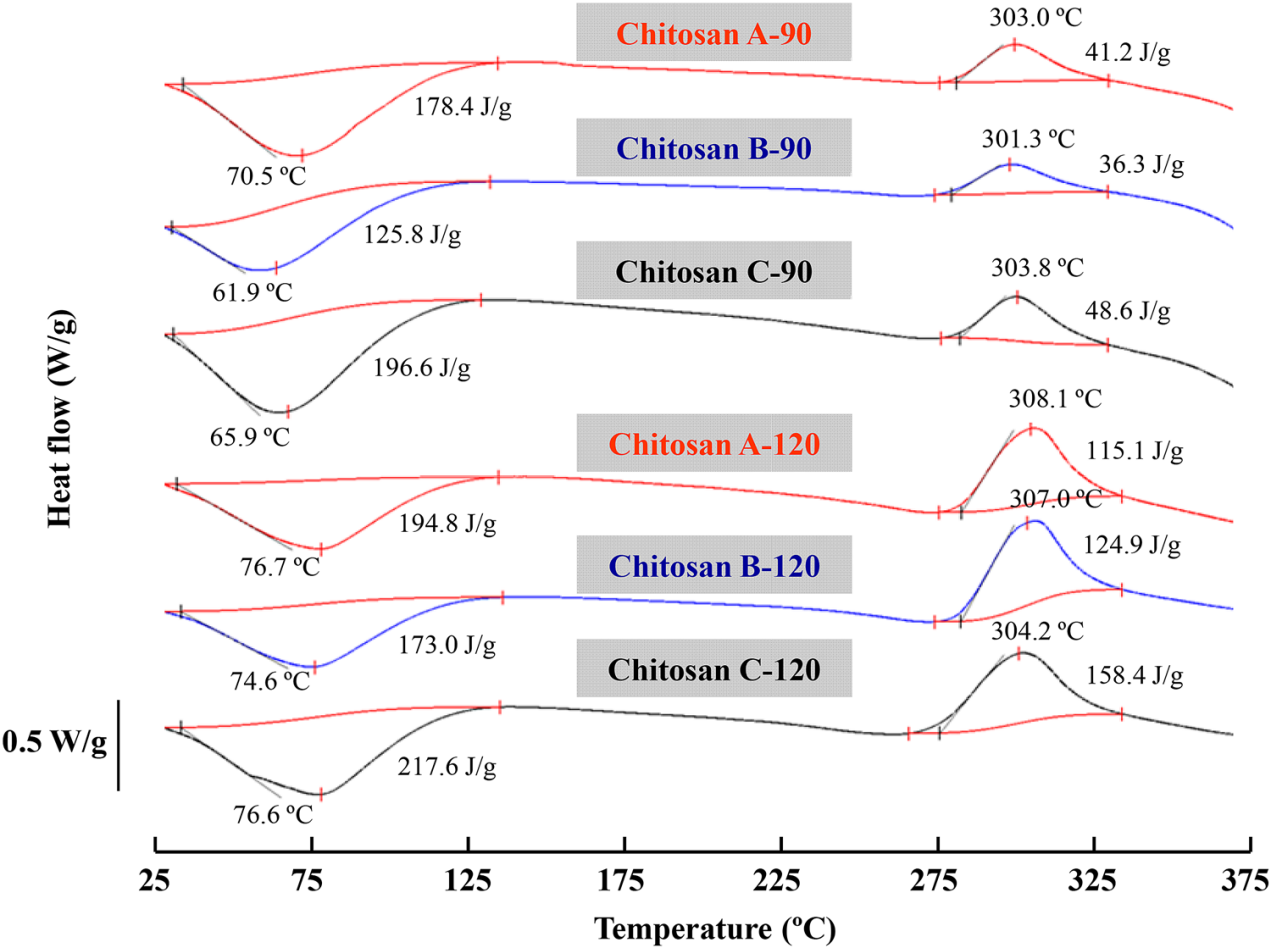
DSC curves of crab shell chitosan by deacetylation at 90 °C and 120 °C.

The thermal stability of chitosan obtained from swimming crab shells was investigated via DSC (Fig. 5). An endothermic peak at 70.5 °C and an exothermic peak at 302.3 °C were observed in chitosan A-90. The endothermic peak was mainly caused by water removal at the heating temperature^34^. The higher the temperature of endothermic peak, the higher the water-holding capacity of chitosan will be^34–36^. Compared with the endothermic peak of the chitosan prepared at 90 °C by NaOH pretreatment, that of chitosan prepared by subcritical water pretreatment was considerably lower, which slightly increased when the subcritical water contained citric acid. A previous study indicated that O- and N- carboxymethyl derivatives of chitin and chitosan showed higher *ΔH* values than chitosans^32^. A similar trend was observed in the exothermic peak of chitosan prepared via different deproteinization methods (Fig. 5). The exothermic peak is associated with the decomposition of chitin/chitosan macromolecule chain^37^. The exothermic peak area and the peak height ascribed to the amine (GlcN) groups increased when chitosan was prepared via thermochemical alkaline deacetylation^37^. Similar to those of the chitosan prepared by deacetylation at 90 °C, the exothermic peak area and the peak height were also observed in the chitosans prepared at 120 °C due to the high DD. However, the difference among chitosans prepared at the same deacetylation temperatures was negligible, suggesting that subcritical water pretreatment did not influence the thermal stability of the prepared chitosan. This could be due to the fact that the prepared chitosan products contain different contents of chitin, chitosan and the derivatives.

### TLC analysis

**Figure 6:**
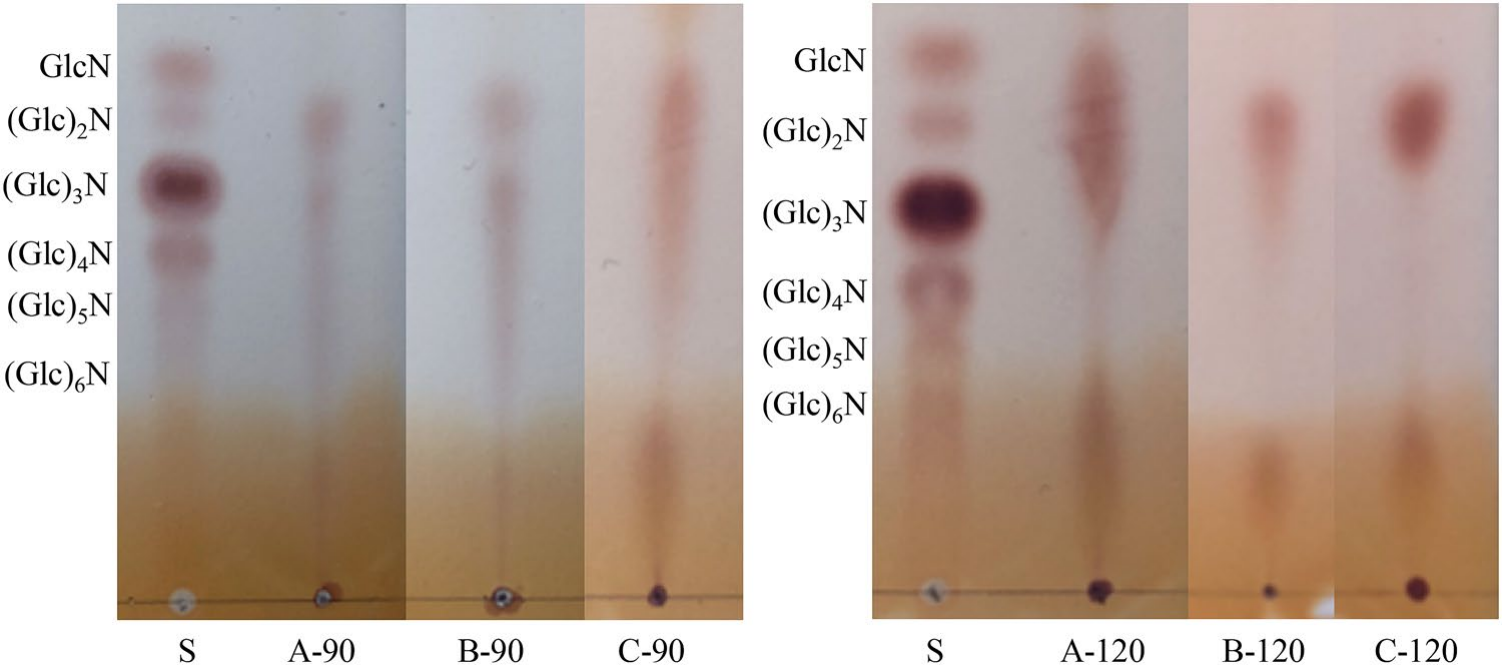
TLC of chitooligosaccharides from crab shell chitosan by deacetylation at 90 °C and 120 °C. S: a standard mixture of chitosan-oligosaccharides.

The TLC of chitosan hydrolytic products digested by chitosanase is shown in Fig. 6. After digestion, the oligosaccharides obtained from chitosan A-90 mainly consisted of chitosan dimers (GlcN)_2_, trimers (GlcN)_3_, tetramers (GlcN)_4_, and pentamers (GlcN)_5_, which were continuously distributed. A similar trend was observed in the TLC of chitosan B-90 hydrolytic products, whereas glucosamine (GlcN) was also observed in chitosan C-90 hydrolytic products, except for those oligosaccharides. When the chitosan prepared by deacetylation at 120 °C was used for digestion, the molecular weights of the prepared oligosaccharides decreased. The hydrolytic products of chitosan A-120 were mainly composed of GlcN, (GlcN)_2_, and (GlcN)_3_, whereas those of chitosan B-120 and C-120 primarily consisted of (GlcN)_2_. Chitosan oligosaccharides cannot be obtained from α-chitin by sub- or supercritical water treatments, whereas (GlcNAc)_2_ can be obtained by subcritical (350 °C) or supercritical water (400 °C) pretreatments followed by enzymatic degradation^7^. Chitosanase has high specificity for cleaving GlcN–GlcN links^38^. Thus, the TLC results (Fig. 6) suggested that the subcritical water temperature of 170 °C caused slight differences in chitosan structure. However, the difference between subcritical water pretreatments without and with citric acid was not obvious.

Chitosan with a DD of approximately 88% can be produced by subcritical water pretreatment (instead of alkali pretreatment) and low alkali concentration (30%) for deacetylation at 120 °C, leading to a reduction in environmental pollution during industrial chitosan production. However, deacetylation is traditionally conducted at an alkali concentration of 40–60% and at 90 °C, in which the normal batch reactor can be used because the temperature is below the boiling point of water. At 120 °C, a high-pressure reactor, such as an autoclave, should be used for deacetylation. In a follow-up study, we will attempt to optimize the deacetylation process for preparing chitosan with high DD at low alkali concentrations and at 90 °C.

## Conclusions

Chitosan from swimming crab shells was prepared via subcritical water pretreatment instead of alkali treatment. Regardless of deacetylation temperature, subcritical water pretreatment promoted deacetylation and decreased the molecular weight of the prepared chitosan. The promotion effect was improved when the subcritical water contained citric acid. UV absorption and FTIR and XRD analyses revealed that the chitosan deacetylated at 90 °C also exhibited the characteristics of chitin. However, changes in the structure of the prepared chitosan were not obvious. As molecular weight decreased, the compactness and thermal stability of the prepared chitosan evidently increased. Compared with the chitosan pretreated with sodium hydroxide, (GlcN)_2_ could be easily prepared by digesting with chitosanase from chitosan deacetylated at 120 °C after subcritical water pretreatment. Thus, subcritical water could be potentially used for the pretreatment of crustacean shells. The residues obtained can be utilized to prepare chitosan/chitosan oligosaccharide-based products.

## Materials and methods

### Materials and chemicals

Crab shells were obtained from YingFeng Food Co., Ltd. (Zhangzhou, China), packed in plastic bags, and stored at − 20 °C. Chitosanase was purchased from Wuhan Wanrong Science and Technology Development Co., Ltd. (Wuhan, China). Silica gel thin-layer chromatography (TLC) plates were provided from Merck (Darmastadt, Germany). All other chemicals used were of analytical grade.

### Chitosan preparation

**Scheme 1:**
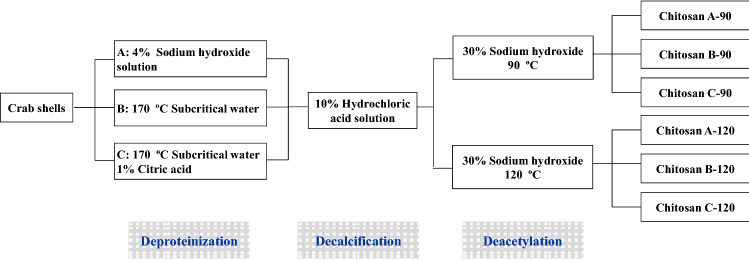
Preparation of chitosan from swimming crab shells.

Chitosan was prepared from swimming crab shells in accordance with Scheme 1. The crab shells were cleaned with tap water and repetitively deproteinized using sodium hydroxide, subcritical water, and subcritical water containing 1% citric acid. (A) Sodium hydroxide was used for deproteinization as follows: crab shells (100 g) were treated with 1500 mL of aqueous sodium hydroxide solution (4%) at 90 °C for 2 h to remove proteins. The deproteinized crab shells were washed with distilled water to neutral pH. (B) Subcritical water was used for deproteinization as follows: crab shells (100 g) and water (1500 mL) were placed in a stainless steel 316 reactor with a volume of 2000 mL, and the water temperature was raised to 170 °C within 30 min. After subcritical water pretreatment at 170 °C for 1 h, the reactor was allowed to cool down to room temperature by flowing water within 30 min. The obtained crab shells were rinsed with distilled water. (C) Subcritical water containing 1% citric acid was used for deproteinization as follows: crab shells were pretreated with subcritical water containing 1% citric acid following the method described in (B) at 170 °C for 1 h.

After deproteinization, the crab shells were demineralized with 10% HCl solution at a ratio of 1:10 (w/v) for 5 h at ambient temperature to prepare chitin. Chitin residues were washed with distilled water to neutral pH and dried in a drying oven at 50 °C. Chitosan was prepared by alkali treatment of chitin by using 30% (w/v) sodium hydroxide solution for 6 h at 90 °C and 120 °C. The residues were washed with distilled water to neutral pH, and the obtained chitosan samples (A-90, B-90, C-90, A-120, B-120, and C-120) were dried at 50 °C for 24 h.

### Yield and ash contents

Chitosan yield was calculated as the dry weight of the obtained chitosan divided by the wet weight of the original crab shells. Chitosan ash content was measured at 550 °C for 6 h.

### DD determination

The DD of chitosan was determined via potentiometric titration. A chitosan sample (200 mg) was dissolved in 30 mL of 0.10 M HCl and diluted to 50 mL with water. The obtained chitosan solution was titrated with 0.10 M NaOH. DD represented the percentage of amino groups in the chitosan calculated from the consumed volume of NaOH solution between two inflection points of acid–base titration^39^.

### Molecular weight determination

The molecular weight of chitosan was determined by viscometry following the method described by Kucukgulmez et al.^12^. Chitosan samples were dissolved in a solvent of 0.2 M NaCl/0.1 M AcOH, and the efflux times of the chitosan solution were measured using an Ubbelohde capillary viscometer at a constant water bath temperature of 25 °C. The running time of the chitosan solution and solvent was recorded in seconds (s) and used to calculate intrinsic viscosity.

### UV absorption spectrum

The UV absorption spectrum of chitosan was measured using a UV-2600 spectrophotometer in accordance with the method outlined by Liu et al.^40^. The chitosan (100 mg) dissolved in 10 mL of 0.1 M HAc solution and UV spectrum was measured between 190 and 400 nm at a scan speed of 2 nm/s with an interval of 1 nm.

### FTIR spectroscopy

FTIR spectroscopy was conducted using a Thermo Nicolet iS50 FTIR spectrometer (Thermo Scientific, Waltham, MA, USA). KBr was used as the diluent and mixed with 1% chitosan powder by using an agate mortar. A transparent troche containing the mixture was obtained by compression with a tableting press. The spectra within the range of 4000–400 cm^−1^ were rationed, and the automatic signals gained were collected in 32 scans at a resolution of 4 cm^−1^ against a background of KBr.

### XRD analysis

Chitosan crystallinity was analyzed using a Philipps X’Pert Pro diffractometer with Cu K*α* radiation (30 kV, 20 mA). Chitosan was prepared by compressing it in the cassette sample holder without any adhesive substances. Data were collected at a scan rate of 5°/min with the scan angle ranging from 5° to 50°.

### Scanning electron microscopy (SEM)

The microstructures of chitosan samples were observed using a Phenom Pro Model desktop scanning electron microscope (SEM, Hillsboro, OR, USA). Prior to SEM analysis, dried samples were conditioned in a desiccator containing silica gel for 1 week at room temperature to obtain the most dehydrated powders. The dehydrated samples were mounted on aluminum specimen holders and coated with gold and palladium and visualized by SEM.

### Differential scanning calorimetry (DSC)

The thermal transition of chitosan was measured by a DSC Q2000 (TA Instruments-Waters LLC Co., New Castle, USA). The dried samples (approximately 1 mg) were accurately weighed into aluminum pans and scanned from 20–380 °C at a heating rate of 10 °C/min. An empty pan was used as the reference. The melting temperature (*T*_m_) and enthalpy change (*ΔH*) were examined from DSC transition curve using the software included in the DSC analyzer.

### Chitosan–oligosaccharide preparation and TLC analysis

Chitosan (1.0 g) was dissolved in 100 mL of 0.1% (v/v) acetic acid solution. The pH of the obtained chitosan solution was adjusted to 5.0 with 1.0 M NaOH, and chitosanase (30 mg) was added, mixed well, and incubated at 45 °C with shaking for 12 h. After hydrolysis, the chitosan hydrolysate solution was adjusted to pH 7.0 with 1.0 M NaOH and terminated in boiling water for 10 min. The chitosan hydrolysate solution was then immediately cooled in an ice bath and centrifuged at 4000 g for 15 min. The obtained supernatant was concentrated to approximately 10 mL and added with tenfold volume of ethanol for chitosan precipitation. The precipitation was converted to chitosan–oligosaccharide powder by drying at 40 °C. The composition of chitosan–oligosaccharide was analyzed via silica gel TLC in accordance with the method developed by Cabrera and Cutsem^41^. The sample and standard were applied onto a TLC plate in a solvent system composed of *n*-propanol:water:ammonia water (7:2:1, v/v/v), and the obtained plate was developed by spraying methanol containing 0.5% (w/v) ninhydrin.

### Statistical analysis

The results were recorded as the means ± standard deviation, and significant differences between groups were tested using one-way ANOVA and Duncan test at *p* < 0.05.
